# Comprehensive assessment of protein loop modeling programs on large-scale datasets: prediction accuracy and efficiency

**DOI:** 10.1093/bib/bbad486

**Published:** 2024-01-03

**Authors:** Tianyue Wang, Langcheng Wang, Xujun Zhang, Chao Shen, Odin Zhang, Jike Wang, Jialu Wu, Ruofan Jin, Donghao Zhou, Shicheng Chen, Liwei Liu, Xiaorui Wang, Chang-Yu Hsieh, Guangyong Chen, Peichen Pan, Yu Kang, Tingjun Hou

**Affiliations:** College of Pharmaceutical Sciences, Zhejiang University, Hangzhou 310058, Zhejiang, China; Department of Pathology, New York University Medical Center, 550 First Avenue, New York, NY 10016, USA; College of Pharmaceutical Sciences, Zhejiang University, Hangzhou 310058, Zhejiang, China; College of Pharmaceutical Sciences, Zhejiang University, Hangzhou 310058, Zhejiang, China; College of Pharmaceutical Sciences, Zhejiang University, Hangzhou 310058, Zhejiang, China; College of Pharmaceutical Sciences, Zhejiang University, Hangzhou 310058, Zhejiang, China; College of Pharmaceutical Sciences, Zhejiang University, Hangzhou 310058, Zhejiang, China; College of Life Sciences, Zhejiang University, Hangzhou 310058, Zhejiang, China; Shenzhen Institute of Advanced Technology, Chinese Academy of Sciences, Shenzhen 518055, Guangdong, China; College of Pharmaceutical Sciences, Zhejiang University, Hangzhou 310058, Zhejiang, China; Advanced Computing and Storage Laboratory, Central Research Institute, 2012 Laboratories, Huawei Technologies Co., Ltd., Shenzhen 518129, Guangdong, China; State Key Laboratory of Quality Research in Chinese Medicines, Macau University of Science and Technology, Macao, China; College of Pharmaceutical Sciences, Zhejiang University, Hangzhou 310058, Zhejiang, China; Zhejiang Lab, Zhejiang University, Hangzhou 311121, Zhejiang, China; College of Pharmaceutical Sciences, Zhejiang University, Hangzhou 310058, Zhejiang, China; College of Pharmaceutical Sciences, Zhejiang University, Hangzhou 310058, Zhejiang, China; College of Pharmaceutical Sciences, Zhejiang University, Hangzhou 310058, Zhejiang, China

**Keywords:** protein loop, loop modeling, deep learning, AlphaFold2, artificial intelligence

## Abstract

Protein loops play a critical role in the dynamics of proteins and are essential for numerous biological functions, and various computational approaches to loop modeling have been proposed over the past decades. However, a comprehensive understanding of the strengths and weaknesses of each method is lacking. In this work, we constructed two high-quality datasets (i.e. the General dataset and the CASP dataset) and systematically evaluated the accuracy and efficiency of 13 commonly used loop modeling approaches from the perspective of loop lengths, protein classes and residue types. The results indicate that the knowledge-based method FREAD generally outperforms the other tested programs in most cases, but encountered challenges when predicting loops longer than 15 and 30 residues on the CASP and General datasets, respectively. The *ab initio* method Rosetta NGK demonstrated exceptional modeling accuracy for short loops with four to eight residues and achieved the highest success rate on the CASP dataset. The well-known AlphaFold2 and RoseTTAFold require more resources for better performance, but they exhibit promise for predicting loops longer than 16 and 30 residues in the CASP and General datasets. These observations can provide valuable insights for selecting suitable methods for specific loop modeling tasks and contribute to future advancements in the field.

## INTRODUCTION

Loops, composed of random coils and turns, are one of the most flexible regions in proteins and are typically crucial for many biological functions [[1, 2]]. These regions have been found to play a critical role in protein-substrate recognition [3–5], protein allosteric regulation [6], enzyme catalysis [7–13], protein conformational state transition [14] and protein–protein (e.g. antigen–antibody) interaction [15], etc. With the rapid development of experimental techniques in structural biology (e.g. X-ray crystallography, NMR and cryo-EM), a vast number of protein structures have been solved [16], constituting the RCSB Protein Data Bank (PDB) [17]. However, due to their high structural flexibility and irregularity, accurate experimental determination of the diverse loop conformations remains a significant challenge [18], and more than half of the proteins in the PDB contain missing fragments that often correspond to loops [19–24]. Computational approaches for flexible loop modeling are an essential complement to the study of loop structures, particularly for structure-based drug design and molecular simulation of functional protein families (e.g. ion channels, enzymes and antibodies) [25, 26], but identifying native-like loop conformations with high computational efficiency would be a long-standing challenge due to the size of the conformational space.

Commonly used loop modeling methods can be roughly divided into three categories: knowledge-based, *ab initio* and hybrid methods. The knowledge-based methods, also known as the template-based methods, have been developed over the past two decades [27, 28] and include widely used approaches such as Prime [29, 30], FREAD [31], MOE search [32], LoopIng [33], Frag’r’Us [34], ArchPRED [35], SuperLooper [36] and SuperLooper2 [23]. These approaches typically rely on a protein template database and employ various biological and physical properties, along with certain scoring functions, to assess potential candidates [37, 38]. Although knowledge-based methods can be accurate and efficient in cases where highly homologous templates are available, they are limited by the diversity of template databases, especially for long loop predictions [2]. The *ab initio* methods typically consist of two primary components: sampling and scoring. These methods utilize physical knowledge to estimate the stable conformations of loops by calculating their conformational free energy minima [1], such as the cyclic coordinate descent (CCD) [39], the robotic-inspired kinematic closure (KIC) [40, 41], the next-generation KIC (NGK) [42], MODELLER [18], Distance-guided Sequential chain-Growth Monte Carlo (DISGRO) [1], GalaxyFill [43], GalaxyLoop-PS2 [44], ModLoop [45], FALC-Loop [46], RCD [47], OSCAR-loop [48], LEAP [49] and SOAP-Loop [50]. These approaches employ broad sampling of the conformational space by varying, for example, the torsional angles of the loop, but computational cost increases exponentially with each additional amino acid residue. Although *ab initio* methods can handle relatively short loops, they are also limited when it comes to longer ones [2].

The hybrid methods, such as CODA [51] and Sphinx [52], which combine the strengths of both knowledge-based and *ab initio* approaches [44], have been proposed to overcome these limitations. With the rapid development of machine learning (ML) and deep learning (DL) techniques [53], these hybrid approaches have shown emerging potential in loop modeling. Nguyen *et al*. [54] first introduced a DL approach for loop prediction by hybridizing the nearest neighbor idea with the convolutional neural network [55–58] or residual network [58]. According to their results [54], this method required over 1.5 CPU h per task and achieved better accuracy in predicting 20 loops of 8-residue length compared to the NGK approach, but exhibited lower accuracy when predicting loops with 12 residues. A reinforcement-learning-based approach named MoMA-LoopSampler was subsequently proposed to increase the sample size, although its effect became increasingly uncertain as the loop length increased [59]. AlphaFold2 [60] and RoseTTAFold [61] have been the focal point of much attention in the realm of protein structure prediction since their release. AlphaFold2 [60] outperformed all other competitors in Critical Assessment of protein Structure Prediction (CASP) 14 by a shocking margin [62, 63]. RoseTTAFold [61] was proposed concurrently, using a three-track neural network for its prediction, and its accuracy approached that of AlphaFold in CASP14 [64]. So far, both methods are still considered to be inadequate for predicting long and flexible regions [64, 65].

Despite recent significant efforts [2], reliably predicting the structures of loop regions remains a formidable challenge. Unfortunately, to our best knowledge, there is a lack of systematic evaluations on the predictive performance of existing methods on loop regions, and no benchmark datasets containing large and diverse data have been made available thus far. Existing datasets suffer from three key shortcomings: (1) they require updating [66], with most test datasets proposed over a decade ago [47, 67, 68], (2) longer loops, especially those exceeding 15 residues, are often ignored [69, 70] and (3) the data coverage and volume are limited, consisting of only ~100 samples and a few protein types [42, 52, 71]. Therefore, evaluations based on these datasets may not adequately reflect actual model performance. Moreover, current evaluation criteria are often simplistic, relying primarily on metrics such as the root mean square deviation (RMSD) between predicted and experimentally determined backbone heavy atoms. Given this, it is crucial to systematically evaluate existing methods using large-scale data and analyze these methods’ performances from multiple perspectives.

In this study, two non-redundant datasets were constructed to provide in-depth coverage and low similarity, denoted as the General dataset and the CASP dataset for short. The General dataset was composed of a total of 10 423 loop structures from 1249 proteins in the PDB database. The CASP dataset included proteins that were sourced from the CASP13 and CASP14 test datasets but were not present in the template database of the knowledge-based methods. This dataset comprised a total of 549 loop structures from 52 proteins. We selected 13 commonly used approaches on protein loops ranging in length from 4 to 69 amino acids for assessment, i.e. two well-known DL approaches (AlphaFold2 and RoseTTAFold), three knowledge-based methods (FREAD, MOE search and Prime) and eight *ab initio* methods (DISGRO, GalaxyFill and Modeller; and CCD, KIC, NGK, Remodel and RML from the Rosetta suite). To systematically assess the predictive performances of these representative loop modeling methods, RMSD, template modeling score (TM-score), success rate, ranking ability, and computing efficiency were calculated and compared. Moreover, the impact of protein class, loop length and residue types on the performance was evaluated to provide an in-depth analysis. This work may provide valuable references and datasets to support the subsequent improvement and application of loop modeling technologies.

## MATERIALS AND METHODS

### Datasets

#### The general dataset

As one of the most important databases in structural biology, the PDB [17] currently contains over 200 000 solved structures. The General dataset was derived from the PDB by PISCES [72] based on the following criteria: the structure solved by X-ray crystallography, resolution < 2.0 Å, *R*-value ≤ 0.25, sequence identity ≤ 10% and no breaks or disorder within the chain. The non-secondary structural regions were defined by DSSP [73], and the loops were identified by DSSP as code ‘C’ or ‘ ’ (space) and the regions connecting secondary structures of at least four residues. Furthermore, the lack of anchor residues (immediately before and after the loop region) or loops with non-standard amino acids were excluded, and the final number of the non-redundant protein loops was 10 423 (from 1249 proteins), with lengths ranging from 4 to 69 residues. Based on the established criteria from previous studies [24, 47], loops are categorized as follows: short (4–7 residues), medium (8–12 residues), long (13–20 residues) and very long (over 20 residues). Consequently, the General dataset comprises 7583 short loops, 2234 medium loops, 524 long loops and 82 very long loops. According to SCOP [74, 75], the General dataset was divided into five classes (all-alpha, all-beta, a + b, a/b, small protein) based on different secondary structural content: all-alpha and all-beta proteins, containing predominantly alpha-helices and beta-strands, respectively; ‘mixed’ alpha and beta classes (a/b) and (a + b), with alternating and segregated alpha-helices and beta-strands, respectively; and small proteins, with little or no secondary structures.

#### The CASP dataset

The CASP dataset derived from the monomer test dataset in CASP 13 [76] and CASP 14 [77] was applied to assess the predictive power of models without templates or with poor template conditions. In accordance with the loop searching protocol employed in the General dataset, the CASP dataset is comprised of 549 loop structures generated from 52 proteins, containing 394 short loops (4–7 residues), 120 medium loops (8–12 residues), 30 long loops (13–20 residues) and 5 very long loops (over 20 residues).

### Loop modeling methods selected for assessment

Given the size of our dataset, processing over 10 000 samples requires the adoption of techniques that can be implemented locally. Consequently, all commonly used methods that can be implemented locally by us were selected for assessment, while the methods exclusively accessible via web servers or constrained by closed-source code were excluded. This led to the selection of 13 loop modeling approaches for assessment in this work, i.e. two well-known DL approaches (AlphaFold2 and RoseTTAFold), three knowledge-based methods (FREAD, MOE and Prime) and eight *ab initio* methods (CCD, DISGRO, GalaxyFill, KIC, Modeller, NGK, Remodel and RML). Knowledge-based methods primarily differ in their template-searching algorithms, whereas *ab initio* methods diverge in their sampling and scoring techniques. Meanwhile, DL methods are distinguished by their model architecture.

#### AlphaFold2 (version 2.1.1)

AlphaFold2, developed by DeepMind [63], has received significant attention for its innovative architecture for end-to-end structure prediction. The architecture incorporates multiple sequence alignments (MSAs) and pairwise features in a joint fashion, along with the introduction of a new output representation and loss function, leading to remarkably accurate predictions. During CASP14, AlphaFold2 achieved first-place honors by demonstrating near-atomic-level accuracy.

#### RoseTTAFold (version 1.1.0)

In 2021, Baker’s lab released RoseTTAFold based on a ‘three-track’ neural network that can model the structure with accuracy comparable to AlphaFold2 in CASP14 [61]. The ‘three-track’ neural network architecture facilitates simultaneous examination of protein sequence patterns, intra-protein amino acid interactions and potential three-dimensional conformations, thereby enhancing the model in comprehending the relationship between a protein’s chemical components and its folded structure.

#### FREAD (version 1.0)

FREAD is a knowledge-based method with length-independent predictive capability [31]. The use of environment-specific substitution scores to evaluate sequence similarity can substantially improve prediction accuracy. Typically, results are generated within 2 Å RMSD.

#### MOE (version 2022.02)

The MOE Loop Modeler module has a *de novo* search algorithm and a PDB search algorithm [32]. The latter is a knowledge-based approach using structures from the PDB. This method identifies suitable loop candidates by matching anchor residue distances and loop sequences, ensuring structural compatibility and diversity. The process involves rigorous checks for sequence compatibility, join geometry, environment clashes and loop uniqueness.

#### Prime from Schrödinger (version 2022.02)

Buildloop is a rapid, knowledge-based loop-building method utilized within the Schrödinger Prime module [29, 30].

#### C‌CD from Rosetta suite (version 3.13)

The Rosetta software is commonly used in the modeling and analysis of protein structures, including protein design, enzyme design and structure prediction of biological macromolecules and macromolecular complexes. CCD is an *ab initio* method in the Rosetta loop modeling module [39, 78]. It presents an algorithm originally developed for inverse kinematics applications in robotics.

#### DISGRO (original version)

DISGRO [1] is an *ab initio* method published in 2014 for loop conformation sampling and prediction based on a chain-growth sequential Monte Carlo sampling strategy.

#### GalaxyFill (version 1.0)

GalaxyFill is an *ab initio* method, which considers the loop closure problem as determining the six torsions to find the roots of the 16th-degree polynomial in one variable [43]. This method allows a small bond and angle flexibility to expand the configuration space, and Monte Carlo minimization is used to enhance local move efficiency.

#### KIC from Rosetta suite (version 3.13)

The robotics-inspired KIC is an *ab initio* method and an essential module in Rosetta loop modeling [40]. The KIC assessed here refers to the fragment-based KIC (officially denoted as KIC with fragments). It utilizes torsion angles of phi/psi/omega in protein fragments to sample torsional degrees of freedom [79], allowing for the fast sampling of large conformational space.

#### Modeller (version 10.2)

Modeller [18] is a popular homology or comparative protein structure modeling software, in which the LoopModel module is often used to obtain better-quality loops. It relies on an atomistic distance-dependent statistical potential of mean force for non-bond interactions, and no homology-derived restraints are used.

#### NGK from Rosetta suite (version 3.13)

NGK [42] is an *ab initio* method in the Rosetta loop modeling module, which combines the intensification and annealing strategies, and yielded a 4-fold increase over standard KIC in the median percentage of sub-Angstrom models across their dataset.

#### Remodel from Rosetta suite (version 3.13)

Remodel [80], a shortcut to the loop modeling tools in Rosetta, is not an algorithm in itself but an alternative, user-friendly executable to utilize CCD or KIC.

#### RML from Rosetta suite (version 3.13)

The Rosetta model_missing_loop (RML) module is a mentioned component for the NGK preprocessing process [81]. The missing loops are modeled using the loop modeling application and the KIC algorithm.

### Evaluation metrics

Five evaluation metrics were utilized to evaluate the accuracy and efficiency of the loop modeling methods, including the RMSD of backbone heavy atoms in the loop region, TM-score of the whole protein structures, success rate, Spearman’s rank correlation coefficient ($\rho$), Pearson correlation coefficient ($r$) and resource consumption.

#### Root mean square deviation

The most commonly used metric is RMSD, which calculates the root-mean-square distance between the corresponding residues after an optimal rotation from one structure to another [82], as shown in Equation (1). Two different RMSDs were calculated through different ways of alignment, i.e. global-RMSD and local-RMSD. Global-RMSD calculates the RMSD of the target loop backbone heavy atoms (N, Ca, C, O) by using the Kabsch algorithm [83] to align the intact protein template structure and the predicted structure, while the local-RMSD corresponds to the RMSD measured after superimposing the target loop region only. Global-RMSD considers not only the accuracy of the regional loop geometrics but also the overall loop orientations in protein.


(1) \begin{equation*} \mathrm{RMSD}=\sqrt{\frac{1}{N}\sum_{i=1}^N{\delta_i}^2} \end{equation*}

where ${\delta}_i$ is the distance between atom $i$ and a reference structure; $N$ is the sum of the number of the backbone atoms in the loop region.

#### TM-score

TM-score [84] overcomes some deficiencies of RMSD by applying different weight factors and is designed to be independent of protein size. TM-score is ideally more efficient in assessing the similarity between two protein structures with different tertiary structures, which could evaluate the effect of loop modeling on the whole protein conformation, which is defined as


(2) \begin{equation*} \mathrm{TM}-\mathrm{score}=\operatorname{MAX}\left[{L}_{\mathrm{Target}}\sum_i^{L_{ali}}\frac{1}{1+{\left(\frac{d_i}{d_{0\left({L}_{\mathrm{Target}}\right)}}\right)}^2}\right] \end{equation*}

where ${L}_{\mathrm{Target}}$is the length of the target protein that other PDB structures are aligned to; ${L}_{ali}$ is the number of the aligned residues; $di$ is the distance between the $i$th pair of the aligned residues; and ${d}_{0\left({L}_{\mathrm{Target}}\right)}=1.24\sqrt[3]{L_{\mathrm{Target}}-15}-1.8$ is the distance parameter.

#### Success rate

The predictive accuracy thresholds of global-RMSD and local-RMSD were defined as 2 and 1 Å for each task, respectively. Any value below or equal to this threshold was considered a successful task. The success rate for each modeling program was calculated based on this standard. A higher success rate for a given task implies a more promising method to obtain the desired results. The local and global success rates, calculated using the two RMSD approaches, were used for the follow-up evaluations.

#### Spearman’s rank correlation coefficient

Spearman’s rank correlation coefficient ($\rho$) was utilized to evaluate the ability of the tested methods to rank items based on the predicted energy terms or output conformations. This measure accounts for both linear and non-monotonic correlations between two variables. In this study, we calculated the rank of energy and the RMSD of output conformations to determine $\rho$. A high $\rho$ value reflects a method’s strong ranking power. Additionally, the top one and top five outputs were compared to the results with the best performance.

#### Pearson correlation coefficient

The Pearson correlation coefficient ($r$) was employed to assess the linear relationship between the energy scores and global-RMSD values for methods that can generate energy scores. A high $r$ value indicates a strong linear correlation between energies and RMSDs for the respective method.

#### Resource consumption

Resource consumption was calculated by counting the average time consumption per task across all programs in parallel with 48 cores on the Intel(R) Xeon(R) Gold 6240R CPU @ 2.40GHz, except for AlphaFold2 and RoseTTAFold in parallel 20 cores on the Intel(R) Xeon(R) Gold 6240R CPU @ 2.40GHz and a Tesla V100SGPU.

## RESULTS AND DISCUSSION

### Comparison of predictive accuracy

**Figure 1:**
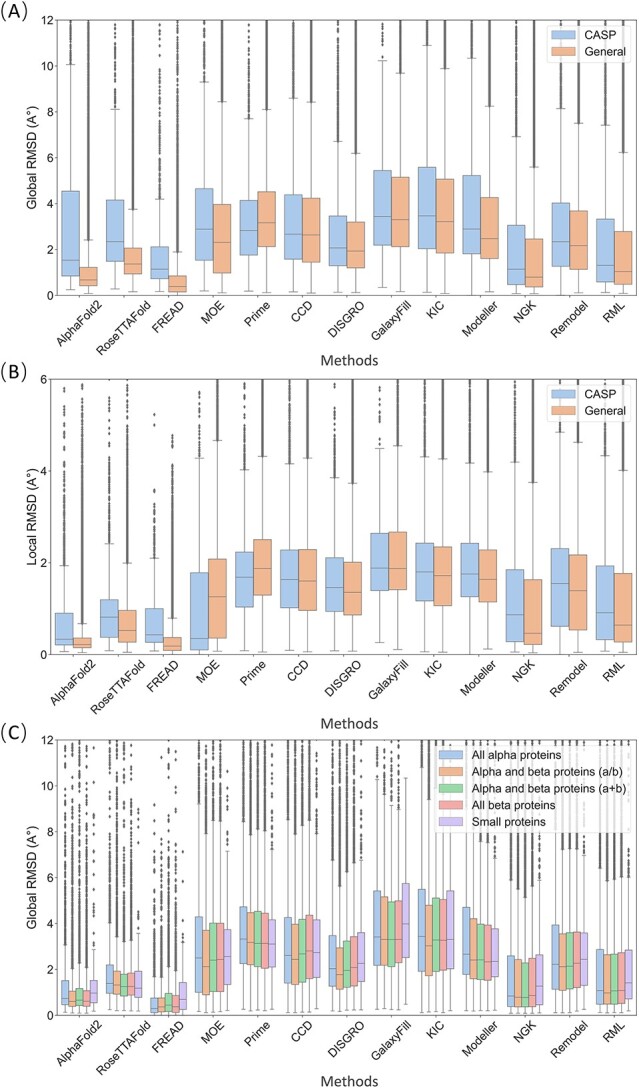
RMSD of the predicted loops by the tested 13 methods on the two datasets. (**A**) Global-RMSD and (**B**) Local-RMSD performance on the CASP and the General datasets. (**C**) The global-RMSD performance on the five secondary-structure classes of the General datasets according to SCOP: all-alpha proteins, a/b proteins, a + b proteins, all-beta proteins and small proteins.

When conducting validation experiments of protein loop modeling, it is necessary to utilize proper metrics to evaluate the reliability of the predicted results. In this work, global-RMSD and local-RMSD were employed as two measures of predictive accuracy. Figure 1A presents the performance of the tested methods in terms of the all-length global-RMSD on the General and CASP datasets. FREAD demonstrated the strongest predictive power among all the tested methods, with a lower global-RMSD and fluctuation than the other methods, on both the General and CASP datasets. Although AlphaFold2 and RoseTTAFold demonstrated commendable performance on the General dataset, exhibiting results relatively comparable to FREAD, their effectiveness was relatively subpar on the CASP dataset. The *ab initio* methods can achieve relatively consistent performance on both datasets, where Rosetta NGK outperformed most tested methods but was still less accurate than the knowledge-based method FREAD. As depicted in Figure 1B, the local-RMSD of a specific loop consistently exhibits lower values than its corresponding global-RMSD, but no significant divergence in the overall trend was observed when assessing the predictive accuracy across different methods. As shown in Figure 1C, the General dataset was further subdivided into five classes (all-alpha, a/b, a + b, all-beta and small proteins), and across the protein classes, FREAD, AlphaFold2 and RoseTTAFold consistently outperformed the other tested methods. Indeed, it is intriguing that the prediction accuracy for small proteins decreased obviously, considering their higher degree of disorder. The poor performance of all-alpha proteins and the improved performance of the a/b class for most methods warrant further investigation.

**Figure 2:**
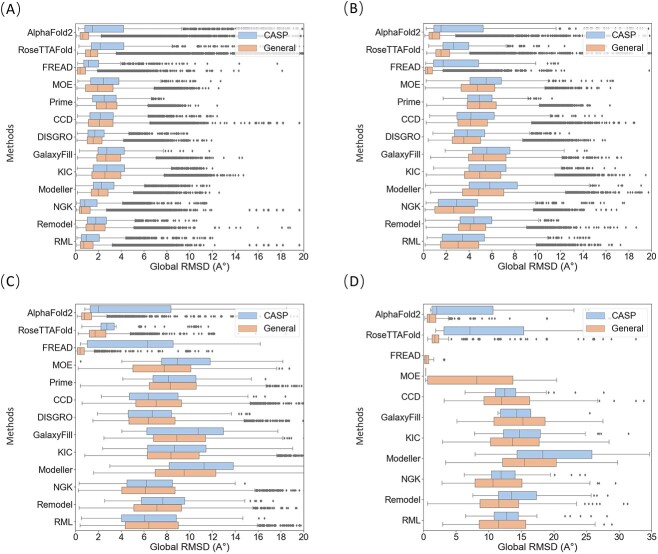
Global-RMSD of the tested 13 methods in different loop lengths on the CASP and the General datasets. Upper box diagram indicates the CASP dataset, lower indicates the General dataset for (**A**) short loops (4–7 residues), (**B**) medium loops (8–12 residues), (**C**) long loops (12–20 residues) and (**D**) very long loops (more than 20 residues).

**Figure 3:**
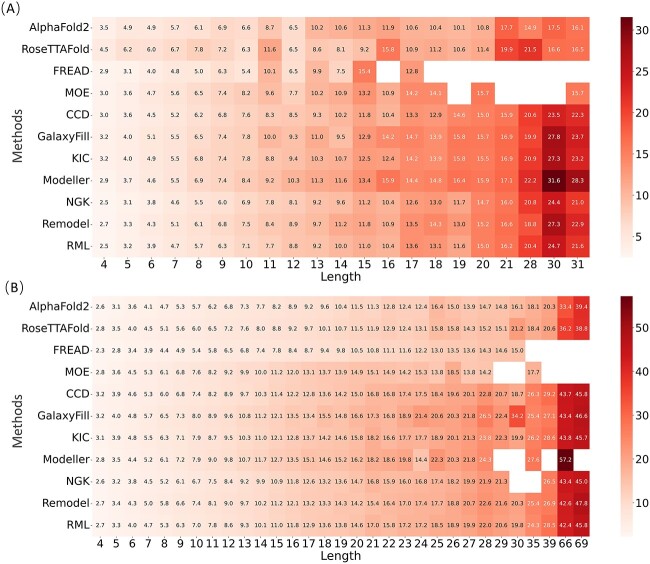
Comparison of the average global-RMSD of different methods at different lengths. (**A**) The CASP dataset. (**B**) The General dataset.

The impact of protein loop length on the predictive accuracy of different methods is further examined. By categorizing loops into short (4–7 residues), medium (8–12 residues), long (13–20 residues) and very long loops (over 20 residues), one may gain insights into how different methods handle loops of varying sizes, as shown in Figure 2. Naturally, all methods performed better on shorter loops (Figure 2A and B) than on longer loops (Figure 2C and D). For the General dataset, FREAD, AlphaFold2 and RoseTTAFold demonstrated remarkable and consistent predictive capacity across all loop lengths, evidenced by median RMSD values of <2 Å, especially with FREAD achieving a median RMSD of <1 Å. However, the predictive performances of these methods noticeably declined for the CASP dataset, especially for loops longer than 12 residues (Figure 2C and D). This could be attributed to the considerable overlap between the General dataset sourced from the PDB and the training data used in DL methods, or the template database used in the knowledge-based methods. Among all *ab initio* methods, NGK exhibited the best, yet the distribution of its results displayed significant dispersion, intensifying with an increase in loop length. Besides, as shown in Figure 2D, FREAD was unable to predict very long loops on the CASP dataset, while DISGRO and Prime failed to process very long loops on both datasets. It could be further observed that the average global-RMSD increased with the rise in loop length (Figure 3), naturally because of the increase in the size of the loops for the RMSD calculation. FREAD showed optimal performance for loops up to 10 and 30 residues on the CASP and General datasets, respectively, but started to show failure in prediction when loops longer than 15 and 30 residues on the CASP and General datasets, respectively. A similar is true for MOE, Modeller and Rosetta NGK, which would also struggle in generating longer loops. DISGRO and Prime seemed to encounter many challenges, which were unable to process prediction starting when loops are around 18-residue lengths. Compared to FREAD, AlphaFold2 and RoseTTAFold generally exhibit rather good performances across all loop lengths on both datasets, especially when loops are longer than 16 and 30 in the CASP and General datasets. Besides, the Rosetta loop modeling methods (including CCD, KIC, NGK, Remodel and RML) exhibit generally comparable performances, where NGK performs exceptionally well in most cases.

**Figure 4:**
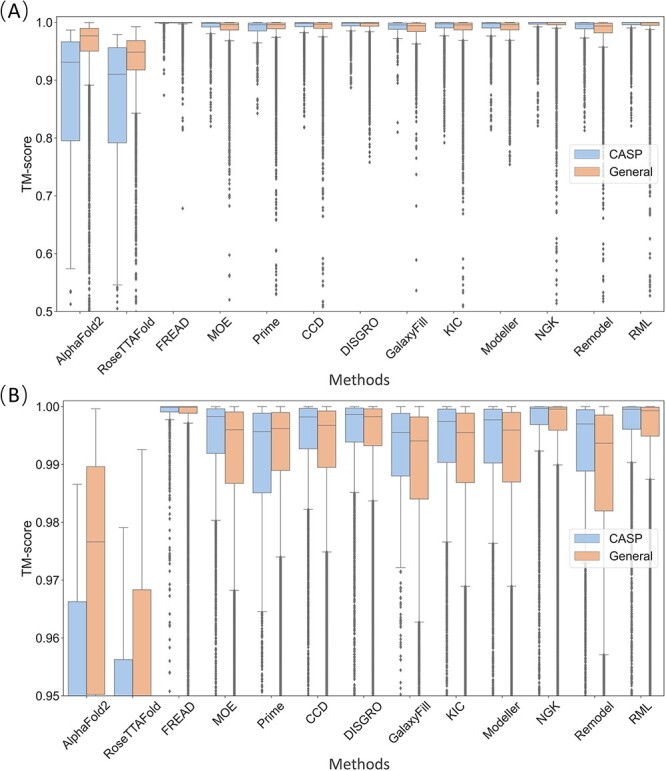
The TM-score of the tested 13 methods. (**A**) The range of values for the TM-scores spans from 0.5 to 1.1. (**B**) The more focused range of values for the TM-scores spans from 0.95 to 1.001.

In addition to RMSD, TM-score was incorporated as an additional criterion for loop modeling assessment (Figure 4). TM-score provides a more comprehensive evaluation of the entire protein structure, including the loop regions. The analysis indicates that the trends in TM-score performance generally align with those of global-RMSD, with the exception of AlphaFold2 and RoseTTAFold, which demonstrate inferior performance. This disparity could be attributed to the fact that these two methods require the modeling of the entire protein structures including loop and non-loop regions, while the other tools exclusively target loop regions, utilizing non-loop structures provided by the user. Among all methods tested, FREAD exhibits the highest degree of proficiency, with NGK emerging as the optimal *ab initio* approach.

### Influence of different amino acid types on model performance

**Figure 5:**
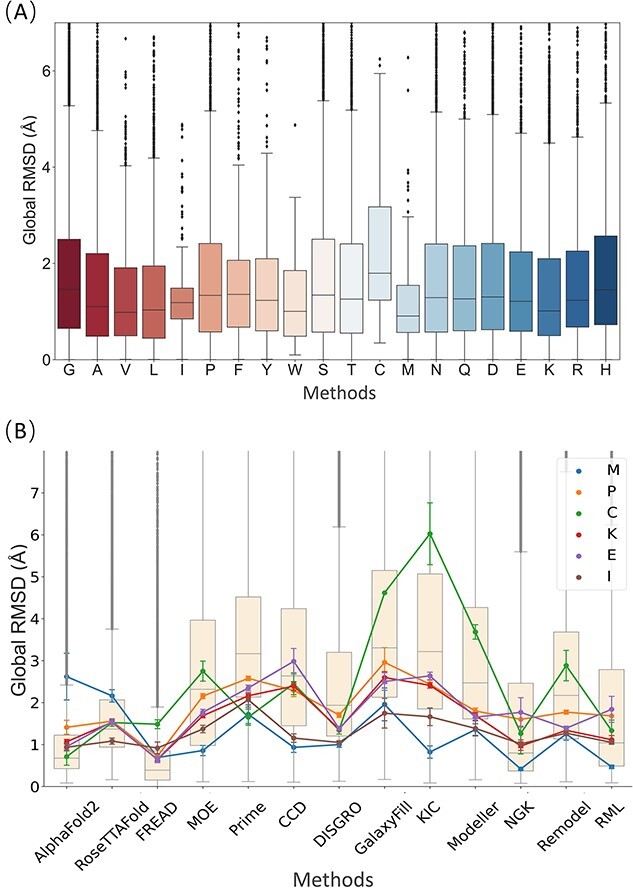
The global-RMSD of loops containing no less than 50% of certain amino acids in the General dataset. (**A**) Global-RMSD of loops containing 1 of the 20 standard amino acids by all tested methods. Loop with non-polar amino acids: glutamic acid (Loop-G), alanine (Loop-A), valine (Loop-V), leucine (Loop-L), isoleucine (Loop-I) and proline (Loop-P); loop with amino acids: phenylalanine (Loop-F), tyrosine (Loop-Y), tryptophane (Loop-W), serine (Loop-S), threonine (Loop-T), cysteine (Loop-C), methionine (Loop-M), aspartic acid (Loop-N) and glutamine (Loop-Q); loop with acidic amino acids: aspartic acid (Loop-D) and glutamic acid (Loop-E); and loop with basic amino acids: lysine (Loop-K), arginine (Loop-R) and histidine (Loop-H). (**B**) Global-RMSD of loops with representative amino acids by the tested 13 methods, shown as the colored dots with error bars. The background box diagram represents the overall global-RMSD performance of the corresponding methods for comparison.

Several studies have reported that the level of protein disorder in the loop regions may be influenced by different amino acids [85, 86]. In this work, the composition of the 20 standard amino acid types in the General datasets was explored. Specifically, we have collected the loops containing no less than 50% of a particular residue type in the General dataset, calculated their global-RMSDs and denoted these loops as the Loop-residue names. For example, Loop-C means a collection of loops in the General dataset containing no less than 50% of cysteine. Figure 5A shows the overall performance of loops containing dominantly each given amino acid generated by all tested methods on the General datasets, where the groups of polar amino acids exhibit the most fluctuation: Loop-C and Loop-M exhibit the worst and best performance, respectively. We further compared the performance of six representative Loop-residues with the overall performance for each tested method (Figure 5B), including nonpolar amino acid types Loop-I and Loop-P, polar types Loop-C and Loop-M, acidic Loop-E and basic Loop-K. In most tested methods, Loop-M demonstrates the best performance, with the smallest mean value and fluctuation, while Loop-C exhibits the largest mean value as well as the dramatic fluctuation across various methods. In AlphaFold2 and RoseTTAFold, however, Loop-M exhibits much worse performance than Loop-C, also dramatically worse than the overall performance. Loop-I contributes much more stable performance than the other Loop-residues, generally better than the overall performance. Loop-P, Loop-E and Loop-K did not exhibit much difference among each other in terms of the global-RMSDs, regardless of the ring or charged group on the side chain. It is seen that the contribution of different residues may vary significantly depending on the programs used, which may help in making informed decisions about which method to use for a specific task or in combination to achieve more accurate predictions.

### Comparison of prediction success rate

**Figure 6:**
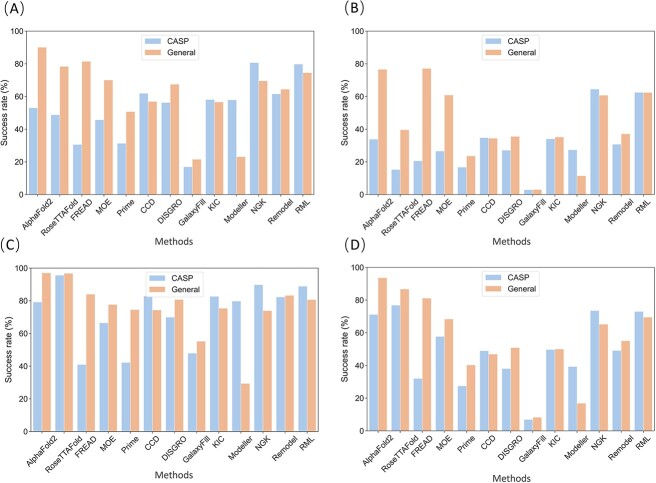
The success rate of 13 methods judged by (**A**) within global-RMSD of 2 Å, (**B**) within global-RMSD of 1 Å, (**C**) within local-RMSD of 2 Å and (**D**) within local-RMSD of 1 Å.

The success rates of the tested 13 methods with different RMSD thresholds are summarized in Figure 6. For most tested methods, roughly, no significant divergence in the overall trend was observed when different thresholds were used, but more detailed analyses would be conducted for more accurate predictions. On the CASP dataset, all knowledge-based methods exhibited lower success rates than *ab initio* methods except for GalaxyFill. Interestingly, RoseTTAFold achieved the highest success rate only when local-RMSD was used as the metrics, while NGK was the best-performing method in terms of global-RMSD, achieving a success rate of 80.69% (cut-off 2 Å) or 64.48% (cut-off 1 Å). On the General dataset, AlphaFold2 demonstrated the highest success rate, achieving 90.1% and 97.1% for global-RMSD (cut-off 2 Å) and local-RMSD (cut-off 2 Å), respectively. As for the knowledge-based methods, the local-RMSD-based success rates were similar to their global-RMSD-based success rates, which is not the case for their performances on the CASP dataset. It further suggests that the variability in predicted results across different programs or methods can be influenced by various factors and metrics, and it would be essential in specific tasks to consider these variations and interpret the results with care. Finally, Galaxy-fill somehow exhibited the lowest predictive accuracy, with success rates below 5% for both global-RMSD and local-RMSD below 1 Å. This might be because Galaxy-fill provided only one loop conformation for each input.

### Assessment of ranking ability

**Figure 7:**
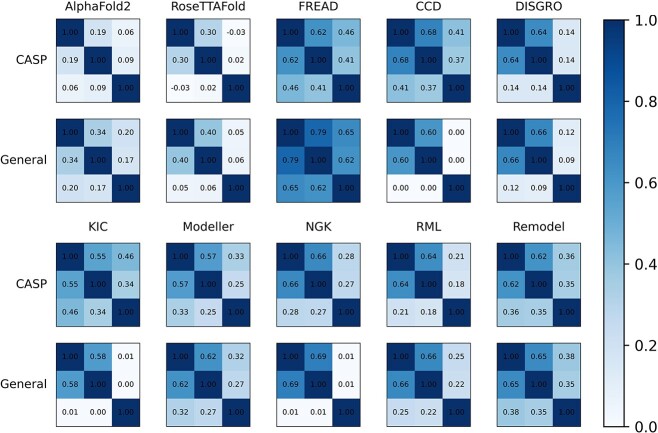
The Spearman’s rank correlation coefficient ($\rho$) between the ranks of global-RMSD, energy and local-RMSD. The columns from left to right and rows from top to bottom of each sub-graph represent the global-RMSD rank, energy rank and local-RMSD rank. The values represent $\rho$.

In practical scenarios, the selection of the most suitable or highly ranked loop conformations is of utmost importance for further study. Accordingly, we computed the Spearman’s rank correlation coefficient ($\rho$) between the ranking results obtained from the energy values calculated by the corresponding program and its RMSD-ordered results, where a larger value of $\rho$ denotes a higher correlation, indicating a stronger ranking ability. For each method tested, we calculated the ranking of each loop conformation based on the global-RMSD, local-RMSD and energy values given by the method. Subsequently, we determined the $\rho$ based on the aforementioned results to evaluate the ranking power of each method. As shown in Figure 7, all the tested methods exhibited a notably higher $\rho$ between the global-RMSD and the energy compared to that between the local-RMSD and the energy. Among these methods, CCD, KIC and NGK from the Rosetta suite demonstrated considerable $\rho$ between the global-RMSD and energy on the General dataset, but almost no correlation was observed between the local-RMSD and energy. Remarkably, FREAD consistently outperformed the other methods, displaying the highest $\rho$ for both the global and local RMSD measurements, indicating its superior ranking capability on both datasets. It is crucial to mention that AlphaFold2 and RoseTTAFold should not be directly compared to the other methods in this context, as they predict the entire protein’s structure and energy. Besides, the distinction between global-RMSD and local-RMSD arises from their respective alignment methods. While global-RMSD relies on the alignment of the entire structure, local-RMSD is computed using the alignment of the loop region alone. Consequently, the $\rho$ value differences derived from global-RMSD and local-RMSD provide insight into the impact of loop orientation on method performance. This suggests that assuming precise loop shape prediction, FREAD demonstrates a reliable capability in accurately predicting loop orientation. Moreover, Table 1 displays the Pearson correlation coefficient ($r$) between the global-RMSD values and energy scores for methods that can offer energy scoring. Notably, Modeller demonstrates the highest linear correlation for the CASP dataset, while NGK is prominent for the General dataset. Importantly, the majority of the methods exhibit more robust linear correlations for the General dataset compared to the CASP dataset.

**Table 1:** Pearson correlation coefficient between global-RMSD and energy score of various methods. *P*-values < 1 × 10^−300^ were represented as 0

Dataset	Methods	Pearson correlation coefficient	*P*-values
CASP	CCD	0.1038	1.83 × 10^−16^
	KIC	0.0690	8.24 × 10^−8^
	Modeller	0.5713	0
	NGK	0.1072	1.43 × 10^−16^
	RML	0.0953	1.13 × 10^−13^
	Remodel	0.1607	1.30 × 10^−25^
General	CCD	0.7360	0
	KIC	0.0041	0.2181
	Modeller	0.5751	0
	NGK	0.7521	0
	RML	0.7453	0
	Remodel	0.3256	0

Table 2 lists the average global-RMSD of the top five and top one conformations sorted by the calculated energies through each method, as well as the top one conformation with the lowest global-RMSD. On the CASP dataset, FREAD, DISGRO, NGK and RML exhibited relatively sound ranking ability, with the average global-RMSD of the energy-top conformations smaller than 3 Å. On the General dataset, FREAD obviously outperformed the other tested methods, achieving the lowest average global-RMSD as well as the standard deviations in all comparisons. AlphaFold2 and RoseTTAFold also gave satisfactory performance on the General dataset, but did not produce consistent results on the CASP dataset.

**Table 2:** Summary of the average global-RMSD of the top five and top one loop conformations sorted by the calculated energies as well as the top one conformation with the lowest global-RMSD

Dataset	Methods	Global RMSD(Å)					
		Top5-energy		Top1-energy		Top1-global RMSD	
		Avg.	Std.	Avg.	Std.	Avg.	Std.
CASP	AlphaFold2	4.20	6.00	4.16	6.17	3.07	4.12
	RoseTTAFold	6.20	12.81	7.50	16.89	4.74	8.20
	FREAD	2.14	2.36	2.56	2.82	2.37	2.84
	CCD	3.21	2.51	3.15	2.43	1.94	1.72
	DISGRO	2.63	2.10	2.53	2.04	1.67	1.30
	KIC	4.22	3.17	4.12	3.12	2.03	1.91
	Modeller	4.01	3.66	3.41	3.68	2.65	3.12
	NGK	2.17	2.63	2.09	2.62	1.22	1.65
	RML	2.38	2.65	2.33	2.57	1.28	1.55
	Remodel	3.04	2.69	2.80	2.66	2.10	1.95
General	AlphaFold2	1.28	2.43	1.16	2.25	0.97	1.95
	RoseTTAFold	1.95	2.73	1.89	2.60	1.71	2.45
	FREAD	0.63	1.08	0.48	1.26	0.39	1.10
	CCD	4.25	6.74	7.93	12.59	2.27	4.62
	DISGRO	2.46	1.97	2.34	1.89	1.56	1.22
	KIC	3.81	2.85	3.78	2.82	1.80	1.67
	Modeller	3.49	3.17	3.52	3.23	2.31	2.55
	NGK	3.35	8.09	8.25	14.85	1.68	5.48
	RML	3.69	8.25	8.81	14.93	1.52	4.12
	Remodel	2.79	2.36	2.77	2.35	1.88	1.74

### Comparison of resource consumption

**Figure 8:**
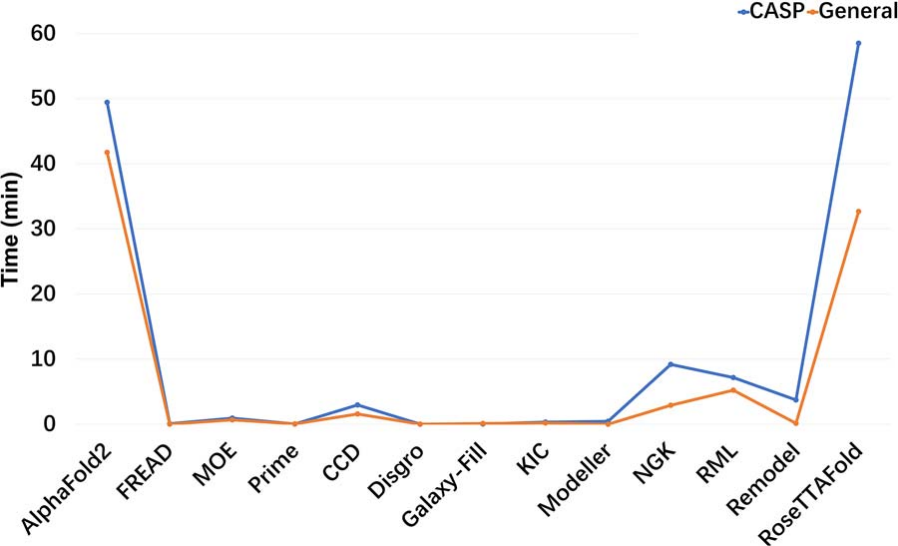
The average time consumption for all the 13 methods based on the General and CASP datasets.

Comparing the resource consumption, especially the time consumption, for different modeling tasks is crucial when dealing with large data volumes. In our comparison, we considered the average time required for each task encompassing AlphaFold2 and RoseTTAFold with 5 conformations, GalaxyFill with 1 conformation and the other methods with 10 conformations. AlphaFold2 and RoseTTAFold were accomplished in parallel with 20 cores on the Intel(R) Xeon(R) Gold 6240R CPU @ 2.40GHz and a Tesla V100S GPU. The other methods were performed parallel with 48 cores on the Intel(R) Xeon(R) Gold 6240R CPU @ 2.40GHz. Since AlphaFold2 and RoseTTAFold predicted the entire protein structure rather than just the loop region, the time consumption of both methods was significantly greater than that of the other methods. The average time consumption of AlphaFold2 was 35.8 min on the General dataset and 49.4 min on the CASP dataset. Similarly, RoseTTAFold required 32.7 min per task on the General dataset and 58.5 min on the CASP dataset (Figure 8). As for the knowledge-based method, FREAD completed the prediction for each task within a few seconds (e.g. 4.56 and 0.84 s on the CASP and the General datasets, respectively). As expected, the *ab initio* methods required more time than FREAD. Most Rosetta loop modeling methods, including CCD, NGK, Remodel and RML, exhibited a consistent pattern of lower time consumption on the General dataset in comparison to the CASP dataset. As an illustration, NGK demonstrated a noteworthy discrepancy, requiring 9.2 min per task on the CASP dataset, which was twice the time taken on the General dataset. Remarkably, Modeller and DISGRO displayed swift execution times at the millisecond level on the General dataset. However, GalaxyFill, despite offering only one conformation, did not manifest superior time efficiency compared to other methods.

## CONCLUSION

In this study, we constructed two benchmarks (i.e. the General dataset and the CASP dataset) and comprehensively assessed 13 loop modeling methods (i.e. AlphaFold2, RoseTTAFold, FREAD, MOE search, Prime, CCD, DISGRO, GalaxyFill, KIC, Modeller, NGK, Remodel and RML) from multiple aspects, including the prediction accuracy in terms of global-RMSD, local-RMSD and TM-score, the impact of a different protein class, loop length, amino acid types on the performance, success rate, ranking ability and resource consumption.

Among the methods tested, the knowledge-based approach FREAD performed the best in terms of prediction accuracy and ranking ability on both datasets. The *ab initio* methods demonstrated consistent performance across both datasets. Notably, NGK had the highest success rate on the CASP dataset taking global-RMSD as metrics, demonstrating its potent predictive capabilities when modeling non-ideal template samples or those that are infrequently represented in databases. In general, FREAD and NGK performed the best in predicting loops with various shorter lengths on the General and CASP datasets, respectively, but may encounter failures in processing longer loops. Several methods face similar problems, including Prime, DISGRO, MOE search and Modeller. AlphaFold2 and RoseTTAFold showed high potential in predicting long loops and complex tasks that other methods fail to address, but their proficiency declines when dealing with shorter loops, as they are incapable of fixing the non-loop regions. In terms of the loops containing different amino acid types, the contribution of different residues may vary significantly depending on the programs used, where polar residues show relatively dramatic fluctuation across various methods. Taken together, the variability in predicted results across different programs or methods can be influenced by various factors and metrics, and it would be essential in specific tasks to consider these variations and interpret the results with care. For future research, it would be beneficial to concentrate on loop modeling for specific protein families. Additionally, DL-based methods may offer a balance between accuracy and computational efficiency. These findings could hopefully provide valuable insights into the development of loop modeling methods and further contribute to the progress of drug design and discovery.

**Key Points**

- Two rational datasets have been established, comprising over 10 000 samples to evaluate the effectiveness of loop modeling approaches.
- A comprehensive evaluation of 13 wildly used methods including three knowledge-based methods, eight *ab initio* methods and two well-known deep learning methods AlphaFold2 and RoseTTAFold.
- Beyond merely assessing the accuracy and efficiency of these methods, the study delves deeper into investigating the influence of loop length, secondary-structure class and amino acid type on the effect of loop modeling.

## Data Availability

The data can be found at https://zenodo.org/records/10043530.

## Author Biographies

**Tianyue Wang** is currently a master student in College of Pharmaceutical Sciences at Zhejiang University under the supervision of Prof. Tingjun Hou and Associate Prof. Yu Kang. Her research interests mainly lie in the area of computer-aided drug design.

**Langcheng Wang** is currently an undergraduate student at New York University. His research interests mainly lie in the area of computer-aided drug design.

**Odin Zhang** is currently a master student in College of Pharmaceutical Sciences at Zhejiang University under the supervision of Prof. Tingjun Hou. His research interests mainly lie in the area of computer-aided drug design.

**Chao Shen** and **Jike Wang** are currently postdoctoral fellows in College of Pharmaceutical Sciences at Zhejiang University. Their research interests mainly lie in the area of computer-aided drug design.

**Xujun Zhang** and **Jialu Wu** are currently PhD students in College of Pharmaceutical Sciences at Zhejiang University under the supervision of Prof. Tingjun Hou. Their research interests mainly lie in the area of computer-aided drug design.

**Ruofan Jin** is currently a PhD student in College of Life Sciences at Zhejiang University under the supervision of Prof. Tingjun Hou and Prof. Ruhong Zhou. Her research interests mainly lie in the area of computer-aided drug design.

**Donghao Zhou** is currently a master student in Shenzhen Institute of Advanced Technology at the Chinese Academy of Sciences. His research interests mainly lie in the area of computer-aided drug design and computer vision.

**Shicheng Chen** is currently a master student in College of Pharmaceutical Sciences at Zhejiang University under the supervision of Prof. Tingjun Hou and distinguished research fellow Peichen Pan. His research interests mainly lie in the area of computer-aided drug design.

**Liwei Liu** is currently a researcher in 2012 Laboratories of Huawei Technologies Co., Ltd. Her research interests focus on artificial intelligence in healthcare and drug design.

**Xiaorui Wang** is a PhD student in State Key Laboratory of Quality Research in Chinese Medicines at Macau University of Science and Technology. His interests include computer-aided synthesis planning, drug discovery and molecular generation.

**Chang-Yu Hsieh** is currently a professor in College of Pharmaceutical Sciences at Zhejiang University. His research interests are mainly in the area of computer-aided drug design.

**Guangyong Chen** is currently a distinguished research fellow at Zhejiang Lab. His research interests are mainly in the area of computer-aided drug design and computer vision.

**Peichen Pan** is currently a distinguished research fellow in College of Pharmaceutical Sciences at Zhejiang University. His research interests are mainly in the area of computer-aided drug design.

**Yu Kang** is currently an associate professor in College of Pharmaceutical Sciences at Zhejiang University. Her research interests are mainly in the area of computer-aided drug design and molecular modeling.

**Tingjun Hou** is currently a professor in College of Pharmaceutical Sciences at Zhejiang University. His research interests can be found at the website of his group: http://cadd.zju.edu.cn.
